# Revisiting the standard for modeling functional brain network activity: Application to consciousness

**DOI:** 10.1371/journal.pone.0314598

**Published:** 2024-12-16

**Authors:** Antoine Grigis, Chloé Gomez, Vincent Frouin, Edouard Duchesnay, Lynn Uhrig, Béchir Jarraya

**Affiliations:** 1 Université Paris-Saclay, CEA, NeuroSpin, Gif-sur-Yvette, France; 2 Cognitive Neuroimaging Unit, Institut National de la Santé et de la Recherche Médicale U992, Gif-sur-Yvette, France; 3 Université Paris-Saclay (UVSQ), Neuroscience Pole, Foch Hospital, Suresnes, France; National University of Sciences and Technology, PAKISTAN

## Abstract

Functional connectivity (FC) of resting-state fMRI time series can be estimated using methods that differ in their temporal sensitivity (static vs. dynamic) and the number of regions included in the connectivity estimation (derived from a prior atlas). This paper presents a novel framework for identifying and quantifying resting-state networks using resting-state fMRI recordings. The study employs a linear latent variable model to generate spatially distinct brain networks and their associated activities. It specifically addresses the atlas selection problem, and the statistical inference and multivariate analysis of the obtained brain network activities. The approach is demonstrated on a dataset of resting-state fMRI recordings from monkeys under different anesthetics using static FC. Our results suggest that two networks, one fronto-parietal and cingular and another temporo-parieto-occipital (posterior brain) strongly influences shifts in consciousness, especially between anesthesia and wakefulness. Interestingly, this observation aligns with the two prominent theories of consciousness: the global neural workspace and integrated information theories of consciousness. The proposed method is also able to decipher the level of anesthesia from the brain network activities. Overall, we provide a framework that can be effectively applied to other datasets and may be particularly useful for the study of disorders of consciousness.

## Introduction

Resting-state functional Magnetic Resonance Imaging (rs-fMRI) is frequently used to capture ongoing neuronal activity [1, 2]. A key aspect of rs-fMRI analysis involves examining fluctuations in acquired signals between different brain Regions Of Interest (ROIs), known as Functional Connectivity (FC). This approach is particularly valuable as it provides insights into the organization of ongoing brain activity. Employing linear latent variable models allows high-dimensional observed FC variables to be related to low-dimensional unseen latent variables. Interestingly, these latent variables can be interpreted as reflecting activity within FC networks [3]. The interpretability of such computational models is valuable for many applications, such as linking the state of consciousness to brain activity. In this context, studying consciousness presents inherent challenges due to its subjective nature. To address this, experimental designs often include various conditions that represent different states of consciousness. Examples of these conditions include different sleep stages [4], varying depths of anesthesia, or the effects of different anesthetic agents [5, 6]. Derived FC measures are indirect markers that have been shown to be effective in inferring related states of consciousness.

In this study, we compare normal wakefulness with altered states of consciousness experimentally induced by different anesthetic conditions. The anesthesia-induced Loss of Consciousness (LoC) was first studied using EEG [7–9]. Anesthetics, like many other pharmacologic agents, have been shown to exert direct effects on neurovascular coupling in addition to their effects on neuronal activity, which in turn may further affect neurovascular coupling through changes in brain activity [10–17]. There is ongoing debate as to the extent to which changes in neurovascular coupling are due to changes in brain activity or to direct effects of the drug on the neurovascular coupling itself. In rs-fMRI, several studies have revealed distinct profiles of altered FC associated with isoflurane and ketamine in Non-Human Primates (NHPs) [18, 19]. Additionally, other research using anesthesia-induced LoC has demonstrated a clear separation between conscious and unconscious states [6, 20, 21]. These findings suggest that FC-driven rs-fMRI analyses are relatively insensitive to the specific anesthetic used and its molecular target. This observation suggests that instead of a bottom-up investigation starting from molecules and cells—which would account for anesthetic-specific effects—it is possible to use an information-processing analysis of FC data to study different levels of consciousness. Accordingly, our goal is to develop an FC-driven framework that employs an interpretable linear latent model to identify brain patterns and related activities associated with conscious or unconscious experiences.

The proposed experiments will be performed on a retrospective rs-fMRI dataset composed of NHPs recorded across states of consciousness by modeling LoC with finely tuned EEG-controlled anesthesia [6, 21]. On this dataset, existing analyses either use unsupervised classification approaches [6, 21] or rely on a priori computational model identification [22]. In both cases, the use of a single brain-wide metric limits the model’s interpretability and may not adequately capture the underlying fine-grained spatial processes. Recently, the neuroscience community has started adopting robust data analysis frameworks that integrate both supervised and unsupervised models. These frameworks aim to exploit as much experimental information as possible. They replace traditional unsupervised methods with factorial model fitting, which generates latent variables that are interpretable by design. By adopting this new analytical paradigm, we aim to automate the extraction of insights from existing data. This approach will not only help confirm known findings but also uncover new patterns and relationships that may have been previously overlooked. Additionally, the use of a more interpretable model is expected to facilitate the exploration of new research questions and applications that were not possible with previous techniques. Our ultimate goal is to provide valuable features that can improve clinical practice and support more accurate and timely diagnoses.

Finally, there are several theoretical frameworks to study consciousness. The two prominent theories are the Global Neuronal Workspace (GNW) [23, 24] and the Integrated Information (II) [25, 26] theories. Initially, a semi-automated approach was used to extend the GNW theory to monkeys using the considered retrospective dataset [21]. In this work, we aim to interpret our results in the context of both theories, with particular emphasis on the GNW theoretical model of conscious access, as this is currently the only model that has been translated to NHPs. The GNW model states that a piece of information becomes conscious when it is available to a widely distributed cortico-cortical network [23, 24, 27, 28]. Although the GNW model of consciousness is not a localizationist approach, key neuroanatomical landmarks such as the prefrontal cortex, the parietal cortex, and the cingular cortex are responsible for broadcasting information to make it globally available [29]. Neuroimaging studies have shown a disorganized GNW in anesthetized monkeys [30, 31], suggesting that anesthesia may induce LoC by reconfiguring cortical dynamics, particularly within GNW nodes. Consequently, in the proposed study, the target ROIs will consist of these cortical nodes.

**Fig 1:**
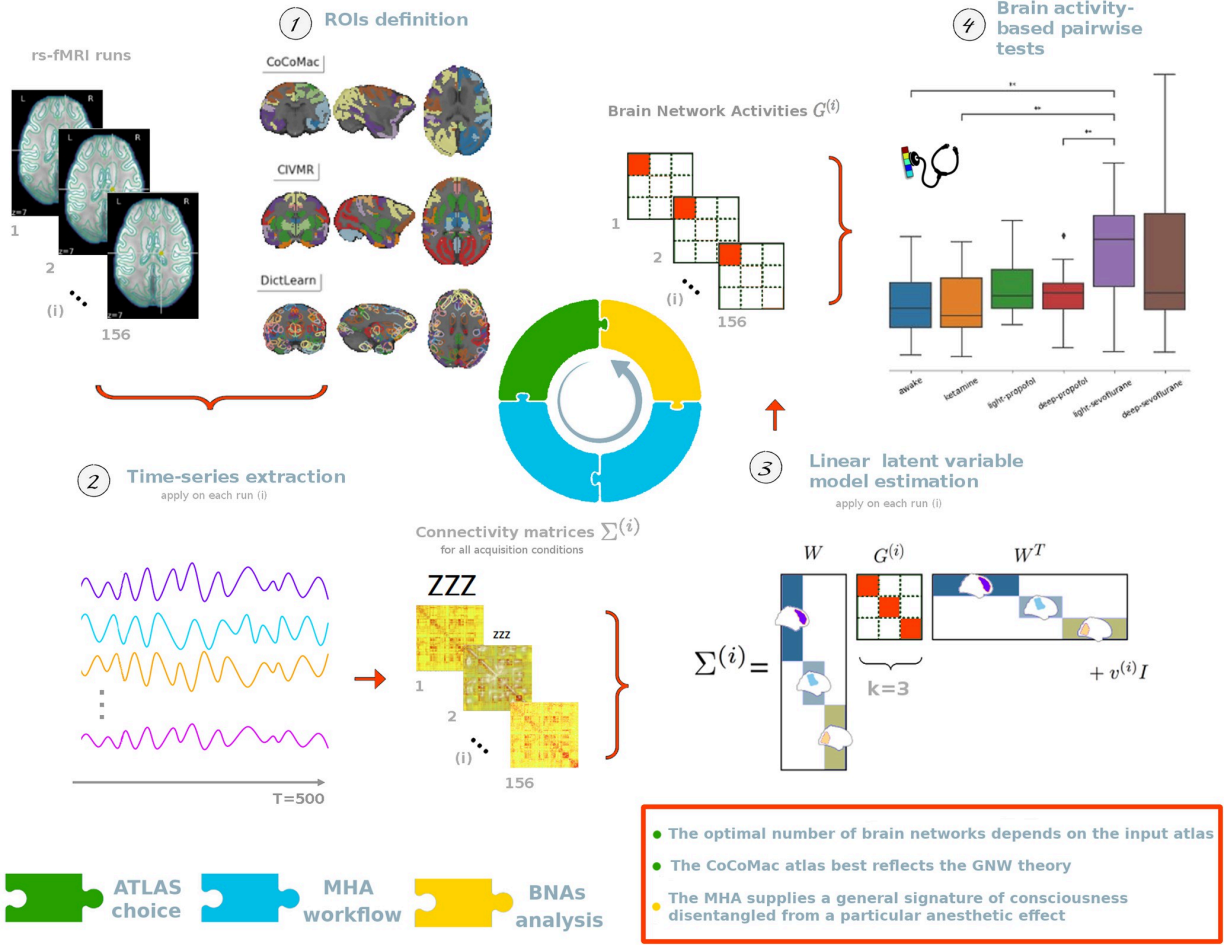
Illustrating the application of the proposed four-step framework to the characterization of different levels of consciousness. Note that the box plot shows the distribution of the functional activities *G*^(*i*)^ that are associated with the brain network 1 for the CoCoMac atlas across all the acquisition conditions. The level of consciousness is indicated by the size of the “ZZZ” notation.

In this work, we develop a computational framework for identifying interpretable spatial signatures of consciousness from rs-fMRI data. The proposed framework characterizes the different levels of consciousness. It consists of four steps (Fig 1): i) generating a list of atlases, ii) filtering and extracting the time series associated with each atlas ROI, iii) recovering disjoint brain networks and associated Brain Network Activities (BNAs) from ROI-based FC matrices that reflect the empirical covariance structures of the data [3], and iv) performing statistical inference and multivariate analysis on the BNAs. The brain networks are disjoint sets of brain regions, and the associated latent variables (the BNAs) form our spatial signatures. As input, standard FC is calculated by averaging time course signals in ROIs, assuming functional consistency within regions. Different atlases can describe these collections of ROIs. However, the functional relationships between them vary from atlas to atlas. At the heart of atlas selection is the question of whether different conditions lead to consistent choices, and whether genericity should be preferred over adaptive strategies. Thus, we extend the work of Monti and colleagues [3] to address the atlas selection problem. In Monti’s seminal work, a strategy is developed to select the optimal number of components/brain networks when decomposing FC structures. In our framework, a machine learning paradigm also provides a sound basis for atlas selection given the underlying clinical question.

The remainder of this manuscript is organized as follows. First, we describe the retrospective dataset studied, including data collected during awake or anesthesia-induced LoC. Then, we provide a detailed explanation of the Modular Hierarchical Analysis (MHA) method and present the considered atlas collection. Finally, we demonstrate that the resulting tailored brain networks and associated BNAs are interpretable. They provide original insights into the study of brain function. Overall, we establish a robust compendium of spatial biomarkers of anestheisa-induced LoC. Among the identified brain networks, one is highly consistent with the GNW. This demonstrates that the MHA can be used in discovery-driven analysis to detect new interpretable signatures of consciousness and anesthesia.

## Materials and methods

### Dataset

This study is a retrospectively analysis of functional imaging data collected nearly 10 years ago in non-human primates in various states of consciousness [6, 21]. The data were acquired in five rhesus macaques (macaca mulatta), one male (monkey J) and four females (monkeys A, K, L, and R), 5 to 8 kg, 8 to 12 years, either in the awake state or under general anesthesia using different molecular agents (ketamine, propofol, or sevoflurane) representing 6 anesthetic conditions. Three monkeys were scanned for each arousal state (awake: monkeys A, K, and J—propofol anesthesia: monkeys K, R, and J—ketamine anesthesia: monkeys K, R, and L—sevoflurane anesthesia: monkeys L, R, and J). For the anesthetics used in this retrospective study, the molecular targets are summarized in S1 Table. Levels of anesthesia were defined by a clinical arousal score (the monkey sedation scale) and continuous EEG monitoring (for details of the anesthesia protocol, see the anesthesia paper [21]). Two different levels of anesthesia are considered for propofol and sevoflurane, either moderate sedation or deep sedation equivalent to general anesthesia, and deep sedation for ketamine. While ketamine is known to induce anesthesia-induced loss of responsiveness that preserves some conscious experiences, at the doses considered, we have previously demonstrated under the exact same anesthesia protocols and doses (inspecting task fMRI [31, 32] or dynamical rs-fMRI [6, 21, 32] consciousness biomarkers) that the resulting brain activity, as assessed by the behavioral scale, achieves anesthesia-induced LoC. 156 rs-fMRI runs (500 volumes per run, TR = 2.4s) were acquired on a 3T Siemens with a customized single transmit-receiver surface coil (see S2 Table for a detailed description of the acquisition conditions distribution across monkeys). The spatial preprocessing is performed by the pipeline described in [21, 33], which includes the following steps: slice timing correction, B0 inhomogeneities correction, motion correction, reorientation, masking, realignment, and smoothing. A run-by-run visual quality control was performed by an expert neuroimager to ensure the quality of the data (i.e. absence of common artefacts, motion, …). The same monkey is implicated in several sessions and runs. Within-session runs may exhibit less variability than between-session runs. Due to legitimate ethical constraints, NHP data are inevitably sparse. Therefore, we independently analyze all 156 rs-fMRI runs acquired under any of the six listed anesthetic conditions. To evaluate this hypothesis, we employ a leave-one-subject-out strategy during the model estimation and inference stages. The goal is to validate the generalization of the model to unseen data. In the following experiments, we leave out J to maximize the number of acquisition conditions in the test set (which consequently excludes any ketamine data from the test set). Alternatively, we could have assessed the consistent performance of the model across subjects, indicating its potential robustness and reliability in different settings. Unfortunately, this approach is not realistic in this retrospective dataset due to the heterogeneity in the distribution of acquisition conditions across monkeys.

The original study was conducted in accordance with the European Convention for the Protection of Vertebrate Animals used for Experimental and Other Scientific Purposes (Directive 2010/63/EU), the National Institutes of Health’s Guide for the Care and Use of Laboratory Animals. Animal studies were approved by the institutional Ethical Committee (Commissariat à l’Énergie atomique et aux Énergies alternatives; Fontenay aux Roses, France; protocols 10–003 and 12–086).

### Establishing a list of atlases

The atlas selection problem is investigated by considering either general reference atlases or a data-driven atlas. General anatomical atlases are defined on other structural or functional datasets, sometimes mixed with histological sections and microscopy. Conversely, data-driven atlases are learned directly from the rs-fMRI data. The former involve the analysis of data based on predefined anatomical or functional regions. The latter are designed to capture the inherent structure and variability of the data itself. While general reference atlases may not fully capture data complexity and variability, data-driven atlases can uncover novel biomarkers and reveal unexpected associations or features. Consequently, a data-driven approach has the potential to identify previously unrecognized functional networks.

Here we consider the CoCoMac atlas, a well-accepted general atlas of the rhesus macaque consisting of 82 cortical regions (41 cortical regions within each hemisphere) [34]. We also consider the CIVMR atlas, which contains a selection of 222 cortical and subcortical regions [35]. To build an atlas based on the dataset, we use the Online Dictionary Learning (DictLearn) from the Nilearn development kit [36]. Using this learning process, we define ROIs directly from rs-fMRI data. A comprehensive analysis of several state-of-the-art strategies recently ranked DictLearn among the best with high robustness and accuracy [37]. DictLearn first masks the data with the cortical/subcortical spatial support of the CIVMR atlas. Next, it extracts connex brain activation regions from the dictionary maps using connected components analysis. We obtain a set of 246 adapted ROIs mapping cortical/subcortical contiguous areas, called the DictLearn atlas. The cross-validation scheme does not evaluate the potential variability of the DictLearn atlas estimation. Thus, the data-driven DictLearn atlas, like the CoCoMac and CIVMR atlases, is considered as prior knowledge of the proposed framework.

### Time series filtering and extraction

Let $I^{\left(i\right)}\in R^{x\times y\times z\times t}$, *i* ∈ [1, *N*] be a rs-fMRI time series volume collected across our cohort of N = 156 experimental runs. *i* refers to complete runs, i.e. multiple observations (statistical samples) of the same (time x space) stochastic process related to the different states of anesthesia. x, y, and z represent the spatial dimension of each volume, and t is the number of time points. Time series denoising operations are applied [6, 21]. Specifically, voxel time series are detrended, filtered with low-pass (0.05-Hz cutoff), high-pass (0.0025-Hz cutoff), and zero-phase fast Fourier notch (0.03 Hz, to remove an artifactual pure frequency present in all the data) filters, regressed out from motion confounds, and z-score standardized. Let *p* be the fixed number of ROIs defined by the atlas. The feature matrix $X^{\left(i\right)}\in R^{p\times t}$ contains the averaged time series computed across all voxels within each ROI. The high number of ROIs in some atlases encourages the computation of the mapping between an atlas and the rs-fMRI data in the high-resolution template space. This way, topology issues induced by the atlas down-sampling are avoided (i.e., a region vanishing due to high contraction in the deformation field). From each feature matrix *X*^(*i*)^, a FC matrix reflecting the data empirical covariance structure is derived and will be analyzed in the sequel.

### Linear latent variable model estimation

#### The Modular Hierarchical Analysis (MHA)

A latent variable model is a statistical model that relates high-dimensional observed variables to low-dimensional unseen latent variables. Such a model can capture complex brain properties that are challenging to quantify or measure directly. From the empirical ROI-based covariance structures or FC matrices, the model estimates a set of distinct brain networks common to all experimental runs, and associated BNAs. This supports the identification of common patterns of brain activity that are consistently observed during different states of anesthesia. The identification of a common brain pattern repertoire across the six considered anesthetic states strengthens the proposed hypothesis [21]. In such a model, the latent variables are the BNAs that are specific to each run *i*. With a limited number of discovered networks, the MHA linear latent variable model has proven to yield more reproducible and explainable results than others such as Principal Component Analysis (PCA) or Independent Component Analysis (ICA) [3]. The MHA approach follows the probabilistic PCA formulation [38]. Briefly, the rs-fMRI observations *X*^(*i*)^ are generated as a linear projection from low-dimensional latent variables $Z^{\left(i\right)}\in R^{k}$. Both observations and latent variables are taken to follow a multivariate Gaussian distribution. We obtain the following generative model for observed data [3]:

$$\begin{array}{c} \begin{array}{cc} Z^{\left(i\right)} & ∼N(0,G^{\left(i\right)})\\ X^{\left(i\right)}|Z^{\left(i\right)}=z^{\left(i\right)} & ∼N(Wz^{\left(i\right)},v^{\left(i\right)}I) \end{array} \end{array}$$ (1)

where $G^{\left(i\right)}\in R^{k\times k}$ is the covariance of latent variables, *k* denotes the number of disjoint brain networks, and $v^{\left(i\right)}\in R_{+}$ is the measurement noise. By capturing the low-rank covariance structure via the shared across-runs loading matrix *W*, the MHA model can reconstruct the covariance matrix in *Σ*^(*i*)^:

$$\begin{array}{c} \Sigma^{\left(i\right)}=WG^{\left(i\right)}W^{T}+v^{\left(i\right)}I \end{array}$$ (2)

$W\in R^{p\times k}$ describes brain networks that are reproducible across the entire population. Each column *j* of *W* encodes the *j*^*th*^ brain network. For each run *i*, the matrix *G*^(*i*)^ contains the latent variables of run *i*. More specifically, the *j*^*th*^ diagonal element of *G*^(*i*)^ estimates the so-called BNA associated with the *j*^*th*^ brain network for run *i*. To compute the model parameters, the optimization maximizes the model log-likelihood L between *Σ*^(*i*)^ and the empirical covariance structure $K^{\left(i\right)}=X^{\left(i\right)}X^{{\left(i\right)}^{\prime }}\in R^{p\times p}$ across all runs as follows:

$$\begin{array}{c} \begin{array}{cc} L(W,G^{\left(i\right)}) & =\sum_{i=1}^{N}p\;log\left(2\pi \right)+log\;det\;\Sigma^{\left(i\right)}+tr\left({\Sigma^{\left(i\right)}}^{-1}K^{\left(i\right)}\right)\\ \hat{W} & =\underset{W:W^{T}W=I;\;W\geq 0}{\text{argmax}}\;L\left(W,G^{\left(i\right)}\right) \end{array} \end{array}$$ (3)

Over PCA, the MHA model adds a non-negativity constraint to the orthonormal constraint. It gives MHA the ability to uncover disjoint brain networks in W and associated run-wise BNAs in *G*^(*i*)^. W has a block structure and is uniquely defined and identifiable. W can be thought of as a shared basis of *k* non-overlapping brain networks across all runs.

As in the work of Monti and colleagues [3], the choice of the optimal number of disjoint networks *k* in the model is treated as hyperparameter tuning. A leave-one-subject-out split is performed to generate a training set and a test set. The MHA model is fitted to the training set, and the log-likelihood L is maximized over the unseen test set. Using unseen data is crucial to evaluate the performance and generalization ability of the model and to avoid overfitting. However, maximizing the log likelihood over unseen data for hyperparameter selection should be done with caution. We know that overfitting can occur critically in our dataset because multiple anesthetic states were administered to the same monkey. To mitigate this risk, we include all of a monkey’s data in the test set.

#### Brain networks matching

For each atlas *l*, *l* ∈ {1 = *CoCoMac*, 2 = *CIVMR*, 3 = *DictLearn*} we get *k*_*l*_ Brain Networks (BNs). In the following, we will compare these networks with a geometric criterion. Let $BN_{i}^{l}$, *i* ∈ [1, *k*_*l*_] be a brain network of a given atlas *l*. $BN_{i}^{l}$ is composed of *N*_*i*,*l*_ ROIs, defined as the number of non-zero values in the *i*^*th*^ columns of *W*^*l*^. The goal here is to match brain networks across atlases. We start by simplifying a ROI (composed of V connected voxels) to its centroid (a 3D coordinate x,y,z). We define the mapping function $\phi :BN_{i}^{l}\in R^{N_{i,l}\times V\times 3}\to {\bar{BN}}_{i}^{l}\in R^{N_{i,l}\times 3}$, where *ϕ* is the mean operator. To compare brain networks between two atlases, we then introduce a proximity measure between a brain network *i* of atlas *l*_1_ ($BN_{i}^{l_{1}}$) and a brain network j of atlas *l*_2_ ($BN_{j}^{l_{2}}$). The proximity metric is defined as the Mean of the Closest (MoC) distance between the set of centroids defined in ${\bar{BN}}_{i}^{l_{1}}$ and ${\bar{BN}}_{j}^{l_{2}}$:

$$\begin{array}{c} \begin{array}{cc} d_{MoC}\left({\bar{BN}}_{i}^{l_{1}},\;{\bar{BN}}_{j}^{l_{2}}\right) & =\frac{d\left({\bar{BN}}_{i}^{l_{1}},\;{\bar{BN}}_{j}^{l_{2}}\right)+d\left({\bar{BN}}_{j}^{l_{2}},\;{\bar{BN}}_{i}^{l_{1}}\right)}{2},\;where\\ d({\bar{BN}}_{i}^{l_{1}},\;{\bar{BN}}_{j}^{l_{2}}) & =mean_{g_{i}\in {\bar{BN}}_{i}^{l_{1}}}min_{g_{j}\in {\bar{BN}}_{j}^{l_{2}}}\|g_{i}-g_{j}\| \end{array} \end{array}$$ (4)

and ‖.‖ is the euclidean norm. Note that the *d*_*MoC*_ proximity metric is symmetric to avoid inconsistencies between sets of different sizes.

### Decoding brain network activities

Only the k-diagonal elements in *G*^(*i*)^ are considered in the proposed analysis. Let $S^{\left(i\right)}=\left(s_{G^{\left(i\right)}}^{1},\ldots ,s_{G^{\left(i\right)}}^{k}\right)$ denote the associated individual activities over the *k* discovered BNs. For all runs *i* ∈ [1, *N*], let $G\in R^{N\times k}$ be the concatenation of the individual activities *S*^(*i*)^. Below we are interested in comparing the different BNAs contained in each column of G: $BNA^{j}=\left(s_{G^{\left(1\right)}}^{j},\ldots ,s_{G^{\left(N\right)}}^{j}\right)$, *j* ∈ [1, *k*]. As such, we can interpret the BNAs as a measure of the activity within the corresponding brain networks. In other words, the BNAs represent the amount of variability carried by each brain network. We hypothesize that the off-diagonal entries of the latent variable covariances are not the most discriminative features for the clinical question under investigation. The non-zero off-diagonal values indicate the presence of redundancy in the data or some degree of correlation between variables.

#### BNA-based statistical inference

Group analysis is performed on the BNAs on an atlas basis to highlight the main discrepancy between anesthetic conditions. Applying the Shapiro-Wilk test reveals that the BNAs do not satisfy the normal assumptions. Therefore, pairwise nonparametric Wilcoxon signed-rank tests are used between paired grouped BNAs. The null hypothesis (H0) states that no significant difference exists between two awake/anesthetized conditions. p-values are adjusted for multiple comparisons using the Benjamini / Yekutieli False Discovery Rate (FDR) correction.

#### BNA-based multivariate analysis

Let the aforementioned $G\in R^{N\times k}$ be the decomposed BNAs, and $y\in Z_{+}^{N}$ the labels encoding the anesthetic conditions (awake state or moderate/deep ketamine, propofol, or sevoflurane anesthesia). Supervised machine learning can predict the outcomes y from the input features G. The proposed classification relies on Support Vector Machines (SVM) with a Radial Basis Function (RBF) kernel (as implemented in scikit-learn [39]). The gamma hyperparameter is automatically determined, while the C hyperparameter is set to 1. To limit overfitting during training in our small-size dataset, bagging is implemented. It aggregates multiple models trained from the base SVM-RBF estimator by randomly taking training subsets. It enables the definition of an overall stronger predictor. As previously described, the model is trained using a leave-one-subject-out splitting to generate a training set and a test set. Model fitting is performed using five-fold cross-validation on the train set. Note that the above classifier treats each class as being non-ordinal and might miss the inherent relationship among the categories to learn. Thus, we also assess the benefit of using another base classification estimator that implements an Ordinal Logistic model with l2 regularization [40] (as implemented in mord [41]). The regularization parameter is set to 1. In both cases, the described hyperparameters are not evaluated in an internal cross-validation.

#### Brain network importance

Finally, it is reasonable to ask which brain networks are involved in the different predictions. Using a model-agnostic permutation importance technique (as implemented in scikit-learn [39]), we measure the importance of features by randomly permuting the values of a feature and evaluating the impact on the model’s performance. By comparing the performance of the model with the permuted features to its original performance, one can determine which features have the most influence on the predictions. Features with a drop in performance after permutation are considered more important. Such a technique helps to identify which features or BNAs and their associated brain networks have the greatest impact on the model’s performance, and can provide insight into the relationships between BNAs and anesthetic conditions.

## Results

### Consciousness-related connectivity can be decomposed into few consistent brain networks

**Fig 2:**
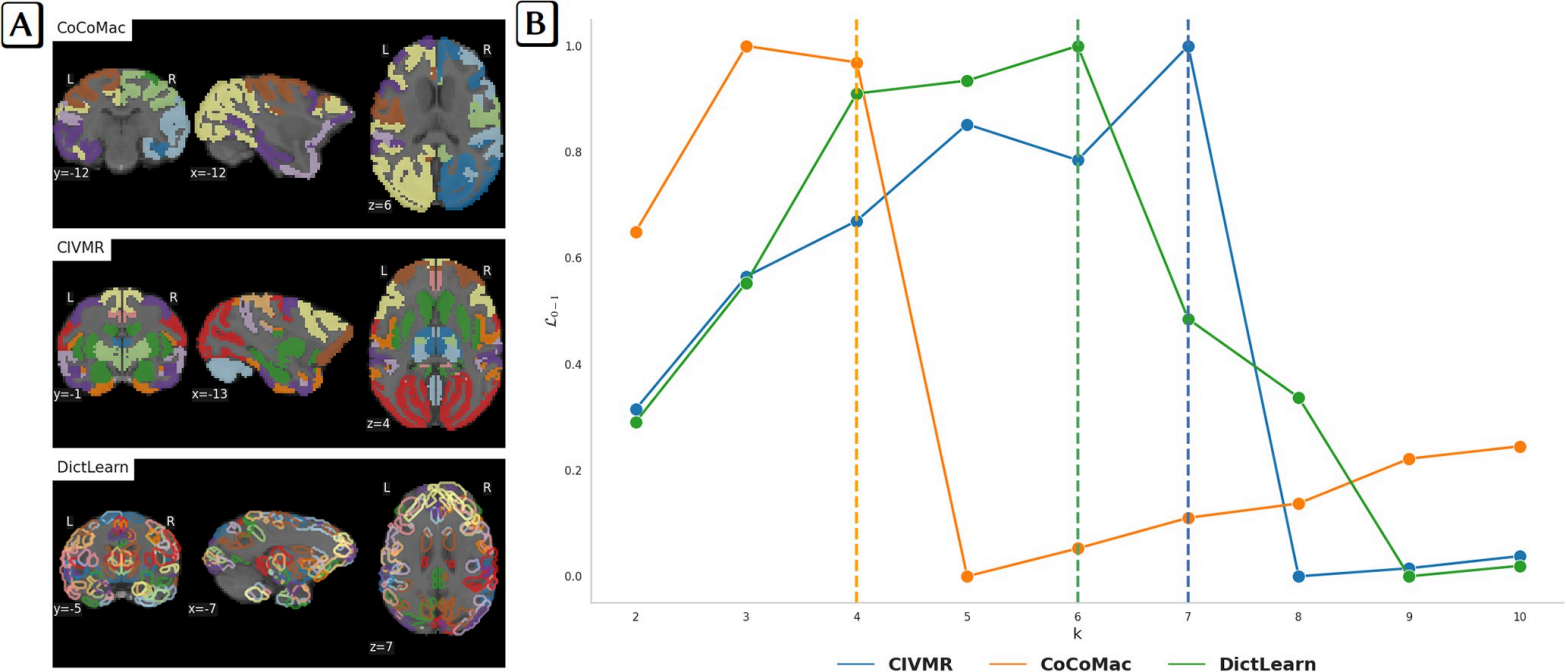
Illustrating how to select the best number of brain networks. A) the CoCoMac, CIVMR, and DictLearn atlases, and B) associated 0–1 normalized log-likelihood $L_{0-1}$ on the unseen validation set for *k* ∈ [2, 10]. The optimal number of networks is represented by a vertical dashed line: k = 4 brain networks for the CoCoMac atlas, k = 6 for the DictLearn atlas and k = 7 for the CIVMR atlas.

**Fig 3:**
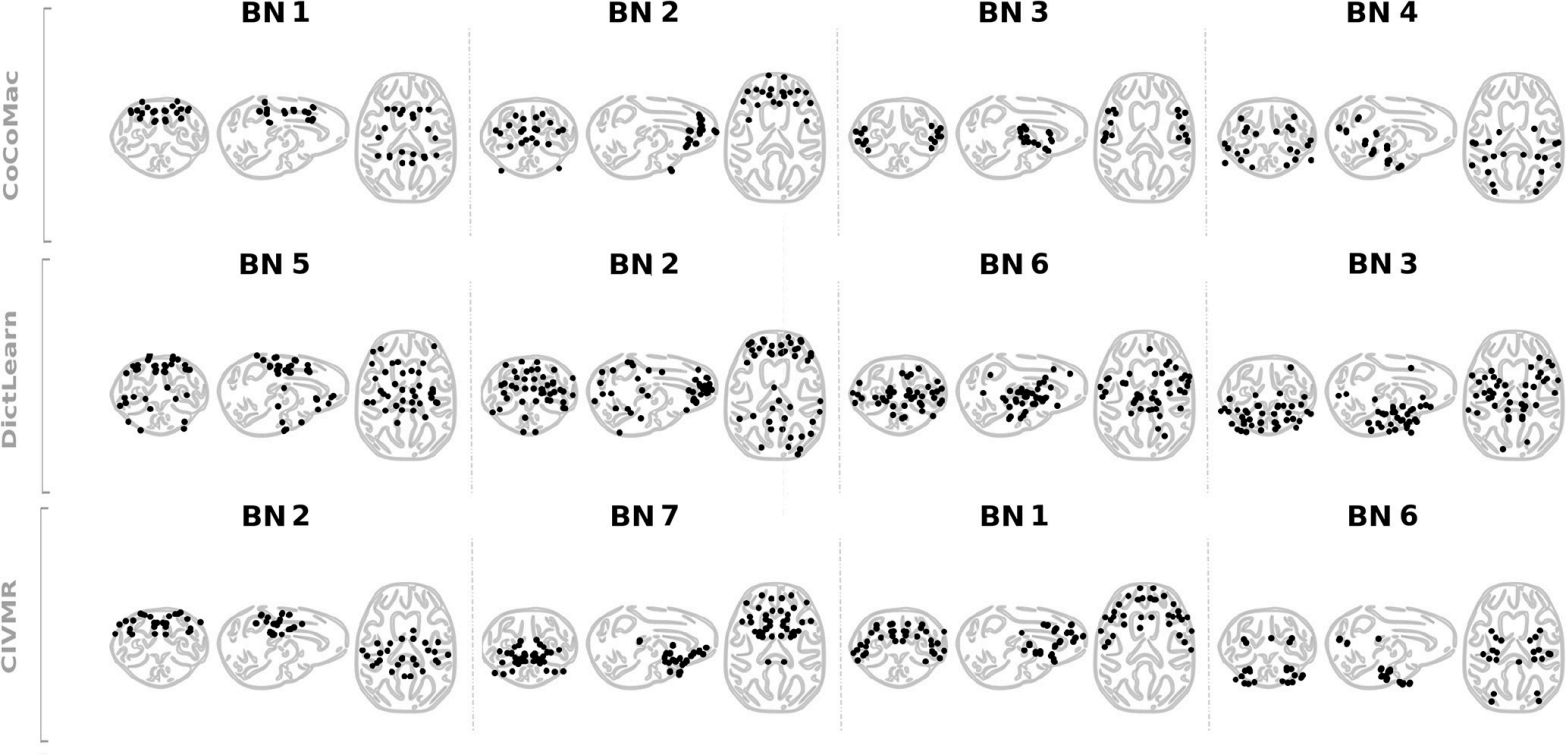
The derived brain networks (BNs). BNs consist of sets of unique ROIs represented by their centroids for the CoCoMac (k = 4), DictLearn (k = 6) and CIVMR (k = 7) atlases. When processing data with different atlases, the resulting BNs are not aligned. Therefore, the BNs are sorted using a geometric criterion (*d*_*MoC*_), and the corresponding BN groupings are displayed in columns. Only the BNs that match in each atlas are displayed. The remaining BNs are listed in S1 Fig.

The choice of atlas controls the number of input regions *p* fed into the model. Maximizing L yields salient *k* = 4, *k* = 6, and *k* = 7 optimal brain networks for the CoCoMac, DictLearn, and CIVMR atlases, respectively (Fig 2). For the CoCoMac atlas, the likelihoods for *k* = 3 and *k* = 4 are very close. We choose *k* = 4 to maximize the number of ROIs included in the resulting BNs (see S3 and S4 Tables for a listing of the selected ROIs for *k* = 3 (66 ROIs) and *k* = 4 (82 ROIs)). Recall that the MHA constraints drive this coverage by conditioning the loading matrix W to have at most one non-zero entry per row and imposing sparsity with non-negativity. For the comparison of the brain networks from the three atlases, the similarity metric *d*_*MoC*_ is applied in a bottom-up fashion. Paired brain networks exhibit shared similarity in their patterns of connectivity (Fig 3). Atlases that provide additional brain networks are globally symmetric (see S1 Fig). It should be noted that by considering all data, we maximize the possibility of obtaining a result that is valid across subjects. Furthermore, given the distribution of acquisition conditions across the monkeys, we felt that this decision to consider all data was justified. Nevertheless, to validate the robustness of the discovered BNs, we re-ran the MHA decomposition using a leave-one-subject-out strategy with the CoCoMac atlas. Interestingly, the generated BNs and BNAs, with a particular focus on BN1 and BN4, exhibit remarkable similarity (see S3 Fig). Furthermore, when listing the differences in terms of the ROIs included in each BN, the differences remain small (see S5 Table).

### Changes in brain networks across states of consciousness

**Fig 4:**
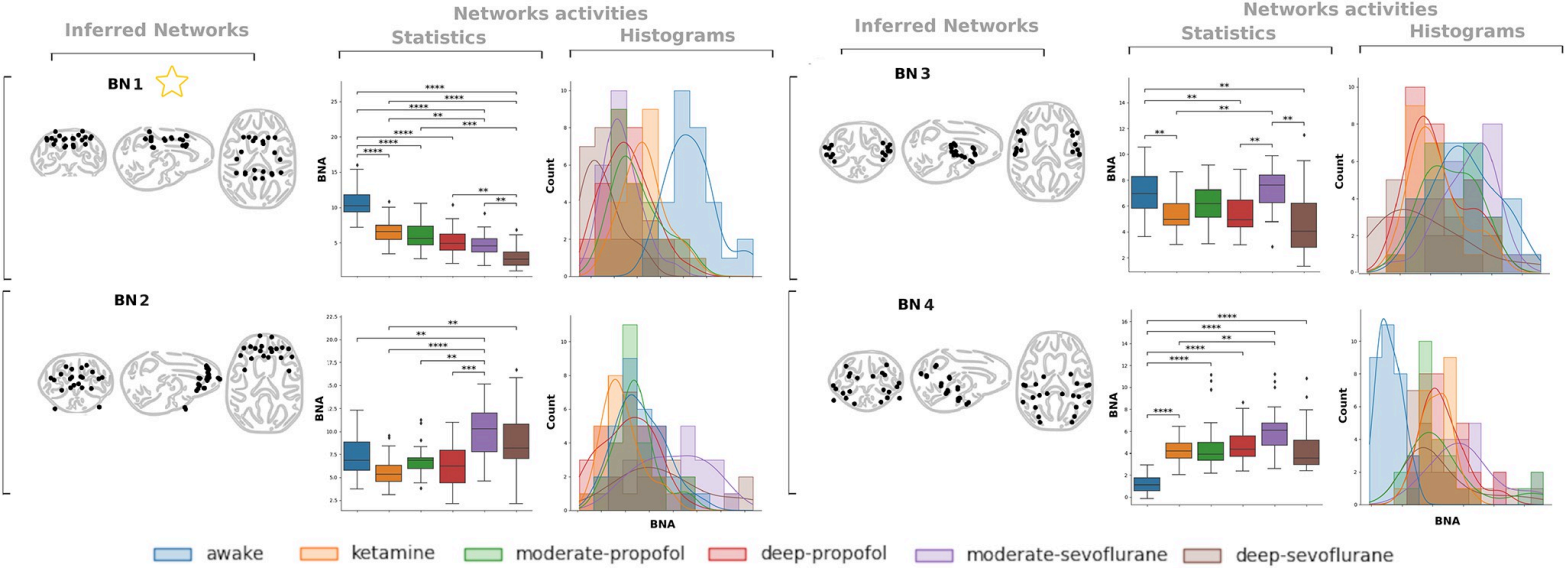
Inferred Brain Networks (BNs) and associated BN activities (BNAs). Four BNs are inferred from the MHA model using the CoCoMac atlas (BN1, BN2, BN3, BN4). Pairwise statistical analysis of the associated BNAs (Statistics) and BNA histograms (Histograms) across different acquisition conditions are presented, in addition to visualizations of the inferred networks (Inferred Networks). The yellow star highlights the brain network (BN1) with a notable difference between the awake state and all anesthetic conditions, which includes the majority of GNW nodes. These plots illustrate how clearly awake can be distinguished from all anesthetic states and, to a lesser extent, how all anesthetic states can be distinguished from each other. The legend for the p-value annotation is as follows: **:1.0*e* − 3 < *p* ≤ 1.0*e* − 2, *** : 1.0*e* − 4 < *p* ≤ 1.0*e* − 3, ****:*p* ≤ 1.0*e* − 4.

In terms of BNAs, the most significant differences between conditions (i.e., states of consciousness) are highlighted using pairwise statistics by preserving the network order (see Fig 4 for the CoCoMac atlas and S2 Fig for the CIVMR and DictLearn atlases). In contrast to the sliding window synchronization patterns [22], our statistics allow for highlighting a larger number of significant differences, with a notable one appearing between the awake state and all anesthetic conditions. The latter result emphasizes the interest of the Brain Network 1 (BN1), which is indicated by a star in Fig 4 and S2 Fig. Interestingly, when focusing on this network for the CoCoMac atlas (BN1 in Fig 4), the ROIs underlying this difference are perfectly symmetric and closely match the macaque GNW nodes [30]: the posterior cingulate cortex (CCp), anterior cingulate cortex (CCa), intraparietal cortex (PCip), frontal eye field (FEF), dorsolateral prefrontal cortex (PFCdl), prefrontal polar cortex (PFCpol) and dorsolateral premotor cortex (PMCdl) areas, which includes the sensory regions of the primary motor cortex (M1), primary somatosensory cortex (S1), primary visual cortex (V1), and primary auditory cortex (A1) (Table 1A). Although obtained in an unsupervised constrained manner, BN1 closely supports the GNW theory (7/11 nodes). The identification of the GNW can only be conclusive on the CoCoMac atlas, as it is the only atlas available to date with the macaque GNW description. To repeat the analysis with other atlases, we should first follow the methodology developed by Uhrig and colleagues [30] to establish atlas-specific GNW nodes. However, we can still utilize the BN alignment metric to discuss the relevance of new related regions identified in the other atlases.

**Table 1:** Listing of Brain Networks (BNs) inferred from the CoCoMac atlas. A) the BN highlighting the difference between the awake state and anesthesia (the BN1 indicated by a star in Fig 4A), and B) the inferred BN4 that is driven by the visual pathway. The detected GNW areas are shown in blue, and the corresponding sensory areas in green.

	name	hemi	location		name	hemi	location
**CCp**	posterior cingulate cortex	left, right	cingulate cortex	**Amyg**	amygdala	left, right	temporal cortex
**CCa**	anterior cingulate cortex	left, right	cingulate cortex	**TCc**	central temporal cortex	left, right	temporal cortex
**S1**	primary somatosensory cortex	left, right	parietal cortex	**TCi**	inferior temporal	left, right	temporal cortex
**PCi**	inferior parietal cortex	left, right	parietal cortex	**PHC**	parahippocampal cortex	left, right	temporal cortex
**PCm**	medial parietal cortex	left, right	parietal cortex	**HC**	hippocampus	left, right	temporal cortex
**PCip**	intraparietal cortex	left, right	parietal cortex	**TCv**	ventral temporal cortex	left, right	temporal cortex
**PCs**	superior parietal cortex	left, right	parietal cortex	**VACv**	anterior visual area (ventral)	left, right	occipital cortex
**M1**	primary motor cortex	left, right	frontal cortex	**V2**	visual area 2	left, right	occipital cortex
**FEF**	frontal eye field	left, right	frontal cortex	**VACd**	anterior visual area (dorsal)	left, right	occipital cortex
**PMCm**	medial premotor cortex	left, right	frontal cortex	**V1**	visual area 1	left, right	occipital cortex
**PMCdl**	dorsolateral premotor cortex	left, right	frontal cortex	**CCr**	retrosplenial cingulate cortex	left, right	cingulate cortex
	(**A**)				(**B**)		

### Which atlas best predicts depth of anesthesia from brain network activity?

**Fig 5:**
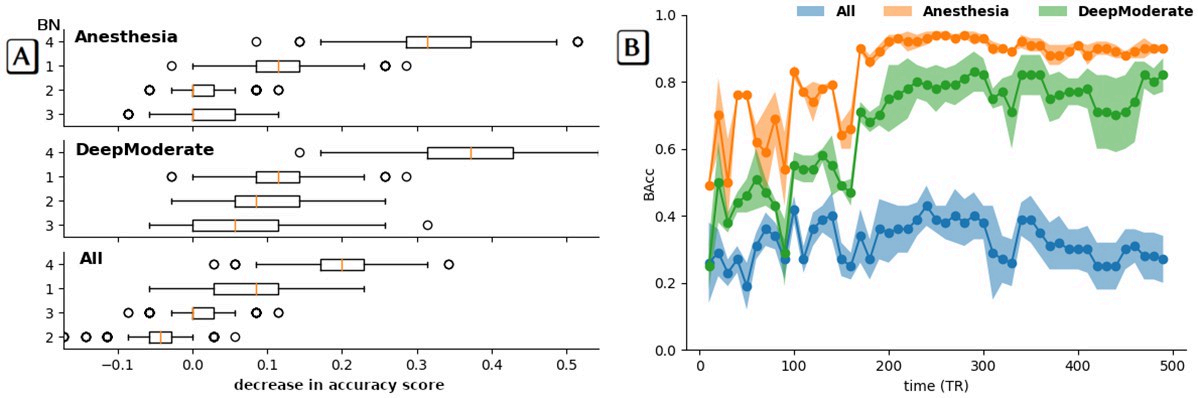
In-depth understanding of how the model works. A) Brain networks (BNs) with the greatest impact on the prediction based on feature of importance analysis for the CoCoMac atlas. Three settings are considered: the awake state and all anesthetics are considered separately (All, theoretical chance level 1/5), the anesthetics are grouped by dosage (DeepModerate, theoretical chance level 1/3), or all anesthetics are encoded in the same group (Anesthesia, theoretical chance level 1/2). Values on the x-axis encode how much model performance decreases with a random shuffling (using balanced accuracy as the performance metric). The amount of randomness is assessed by repeating the process several times. Strong positive values indicate features of interest, while negative values indicate predictions that are more accurate than the real data. The latter can happen when the feature is not important, but randomness makes the predictions more accurate. This is a known behavior with small datasets, which are more prone to random error. B) Learning curves with respect to the acquisition time for the CoCoMac atlas. The Balanced Accuracy (BAcc) metric is used to evaluate the performance of the SVM RBF model. For reasonable performance we need at least 200 TR.

By examining the BNA distributions of BN1 and BN4, we can clearly distinguish the state of wakefulness from the state of anesthesia, regardless of the anesthetics administered to suppress consciousness (Fig 4). Further differences between anesthetics remain. Therefore, we perform a multivariate analysis of the BNAs to establish a decision rule for the atlas selection problem, taking into account the downstream anesthetic state classification task. To further explore BNAs, SVM-RBF and Ordinal Logistic models are considered for solving three classification tasks, corresponding to three sets of target labels: 1) the awake state and each anesthetic are considered separately (label set name: All), 2) the anesthetics are labeled by sedation level (label set name: DeepModerate), or 3) all anesthetics are encoded in the same group (label set name: Anesthesia). For the CoCoMac, CIVMR, and DictLearn atlases, BNA-driven predictions are listed in Table 2. The CoCoMac atlas performs best on the DeepModerate and Anesthesia tasks, demonstrating its relevance in characterizing the depth of anesthesia. It remains competitive to predict the conditions separately. In the following experiments, the SVM-RBF is retained as it gives better performance. Our goal now is to provide more insight into the brain networks discovered with the selected atlas. A brain network importance analysis promotes two brain networks (Fig 5A). Second, BN1, mostly parieto-cingular, is described in the previous paragraph and contains most of the GNW nodes. First, BN4 (Table 1B), primarily located in temporo-parieto-occipital region (posterior brain), contains the visual pathway and may correspond to the fact that awake monkeys have open eyes with potential visual stimulation. Interestingly, BN4 appears also to be interpretable within the II theory of consciousness.

**Table 2:** Brain activity based prediction of acquisition conditions using the CoCoMac, CIVMR and DictLearn atlases.

	CoCoMac			CIVMR			DictLearn		
**SVM RBF**	**All**	**DeepModerate**	**Anesthesia**	**All**	**DeepModerate**	**Anesthesia**	**All**	**DeepModerate**	**Anesthesia**
train	0.76 ± 0.06	0.84 ± 0.02	1.0 ± 0.0	0.8 ± 0.02	0.89 ± 0.03	0.97 ± 0.02	0.8 ± 0.04	0.88 ± 0.01	1.0 ± 0.0
validation	0.58 ± 0.09	0.78 ± 0.14	0.99 ± 0.012	0.61 ± 0.11	0.77 ± 0.08	0.96 ± 0.06	0.71 ± 0.1	0.79 ± 0.02	0.99 ± 0.01
test	0.24 ± 0.05	0.84 ± 0.05	0.97 ± 0.03	0.39 ± 0.08	0.54 ± 0.04	0.76 ± 0.03	0.44 ± 0.11	0.65 ± 0.08	0.75 ± 0.0
**Ordinal Logistic**	**All**	**DeepModerate**	**Anesthesia**	**All**	**DeepModerate**	**Anesthesia**	**All**	**DeepModerate**	**Anesthesia**
train		0.7 ± 0.02	1.0 ± 0.0		0.63 ± 0.05	0.9 ± 0.03		0.79 ± 0.02	1.0 ± 0.0
validation		0.73 ± 0.08	0.99 ± 0.02		0.56 ± 0.07	0.85 ± 0.21		0.75 ± 0.08	1.0 ± 0.01
test		0.79 ± 0.09	0.98 ± 0.01		0.63 ± 0.03	0.68 ± 0.04		0.67 ± 0.05	0.83 ± 0.02

The Balanced Accuracy (BAcc) metric is used to evaluate model performances. Three settings are considered: the awake state and all anesthetics are considered separately (All), the anesthetics are grouped by dosage (DeepModerate), or all anesthetics are encoded in the same group (Anesthesia). Two models are evaluated: the SVM RBF and Ordinal Logistic models. The CoCoMac atlas performs best on the DeepModerate and Anesthesia settings (blue). It remains difficult to predict all conditions, but the CIVMR and DictLearn atlases give the best performance (orange). In this case, the difference with the CoCoMac remains weak.

### Sensitivity analysis of brain activity-driven predictions

Deciphering the level of consciousness from neural activity holds important potential for clinical application, i.e. the development of novel tools to objectively monitor the depth of anesthesia. To investigate the versatility of the method, we evaluate its learning curve as a function of the acquisition duration. This experiment is performed in the case of the CoCoMac atlas, using the three different labeling settings described in the previous paragraph: All, DeepModerate, and Anesthesia (Fig 5B). The duration of the truncated time series ranges from 10 to 500 TR by steps of 10 TR increment. Despite the small dataset size, these plots suggest that a 200 TR run length gives accurate performances (∼0.8 balanced accuracy for the Anesthesia setting). Thus, the proposed solution needs 200 TR to make reliable predictions. This learning curve analysis for a given time series duration is the only practical way to determine a steady state for the model. Although more data would be necessary to confirm this result, it provides an order of magnitude of the minimum buffer size for such an approach.

## Discussion

In the present study, we developed a novel approach for the analysis of rs-fMRI data obtained during different states of consciousness in macaque monkeys. We could extract anatomically relevant cortical brain networks that underlie different states of consciousness. The novelty of the framework lies in the adoption of a constrained linear latent variable model that provides BNAs over identifiable and disjoint ROIs, called brain networks. This new approach allows the construction of an interpretable brain decoding model that provides a unique signature of anesthesia-induced LoC. Within the brain activity recordings, we highlight specific properties (the BNAs) that predictably differ between conscious and unconscious states and that could be used to accurately and objectively classify individuals according to their state of consciousness. A BNA-driven prediction paradigm also ensures that the selected atlas is well suited to investigate the underlying clinical question and provides a sound empirical basis for solving the optimal atlas selection problem.

### Comparison with previous work

Using the proposed dataset, our group has previously described two brain signatures of anesthesia-induced LoC [6, 21, 22]. The first signature is revealed by studying whole-brain dynamics from the rs-fMRI signal. Unsupervised clustering of the dynamic FCs reveals distinct functional connectivity patterns, also called brain patterns. A comprehensive brain signature of anesthesia-induced LoC is provided by the relative prevalence of these ranked brain patterns to a template structural connectivity in the different awake or anesthetized acquisition conditions. As consciousness is lost, the functional connectivity between brain regions becomes increasingly similar to the structural connectivity pattern. This signature was replicated on the same dataset but using a more in-depth connectome harmonics decomposition [42]. Both studies highlight the important role of brain dynamics by examining the dynamic functional organization, but does not attempt to identify subnetworks associated with the identified signature. Alternatively, a second signature is identified by examining the frequency of synchronization levels present in the rs-fMRI signal. Such an analysis of synchronization patterns provides a physiological proxy for the level of consciousness, but has shown poor detection ability. In both cases, we believe that a single brain-wide metric limits the interpretability of the model and is probably inappropriate for modeling the underlying fine-grained spatial processes. The strength of the proposed framework lies in the use of a linear latent variable model that allows interpretation of the latent variables in terms of brain activity within functional sub-networks, and the proposal of an end-to-end framework that addresses the atlas selection problem, statistical inference, and multivariate analysis of the obtained brain network activities. BNAs derived from the MHA model provide reliable biomarkers of anesthesia-induced LoC. Recent work has focused on capturing the dynamics of spatiotemporally overlapping functional networks encoded in the dFCs. Training the MHA model on the dFCs will be the subject of dedicated work. The results will be compared with those obtained by conventional dynamic FC analysis or co-activation pattern analysis such as the iCAPs [43]. Such a comparison would reinforce the unique insights provided by the MHA approach.

### Limitations

#### The optimal number of sub-networks

fMRI neuroimaging and neurophysiological maps provide unique data on brain activity, providing an excellent opportunity to build a whole-brain computational model of LoC. We use such a data-driven machine learning model, the MHA. This model scales to any dataset, and the associated hyperparameters are tuned numerically from maximum likelihood estimation. Specifically, a four-step framework generates a coherent, interpretable, and robust model of consciousness. The discovered brain networks are tailored, spatially consistent, and symmetric. The optimal number of brain networks depends on the input atlas, but a clear decision can be made by monitoring the log-likelihood. Stability analyses of resting-state networks in humans often suggest that 7 to 17 networks are appropriate [44]. In the macaque, ICA allows the detection of 11 prominent resting-state networks involving multiple levels of neural processing that show a remarkable degree of similarity to the human organization [45]. Here, following the seminal work of Monti and colleagues in humans [3], the number of optimal brain networks obtained for the three considered atlases varies from 4 to 7. Capturing the functional organization with few BNs clearly increases interpretability. The question of whether this organization is adequately captured is challenging and will be the subject of dedicated work.

#### A successful benchmark limited by overfitting

As input, we used a retrospective rs-fMRI dataset acquired in macaque monkeys in different states of consciousness, where key results regarding the network organization are expected [6, 21]. The potential of the proposed framework is demonstrated by modeling brain activity on this dataset. Because fMRI acquisition in monkeys is typically performed on a small number of subjects, there is a risk of overfitting. Overfitting occurs when a model becomes too complex and starts to fit the noise or random fluctuations in the training data, rather than the underlying patterns or relationships that are of interest. When working with datasets with a small number of individuals, overfitting can be particularly problematic because there may not be enough examples to capture the true distribution of the data. The small sample size can thus limit the statistical power of the study, making it more difficult to detect true effects and increasing the risk of false negatives. Here, the use of a linear latent variable model is less prone to overfitting than more complex models. A leave-one-subject out strategy is also used in the inference stage. However, it is important to note that the effectiveness of a linear model in limiting overfitting depends on the characteristics of the data and the complexity of the underlying relationships. Increasing the size of the dataset is a common and effective strategy to reduce overfitting in machine learning, typically requiring access to larger datasets. However, given the scarcity of NHP datasets, it may be beneficial to expand the dataset size without additional acquisitions by exploring the advantages of including simulated data [46, 47]. The MHA model, coupled with a rich simulated repertoire of functional brain configurations, would provide a unique framework for exploring artificial LoC in silico and other potential target areas described in different theoretical frameworks (such as the II theory). Note that it is not easy to externally validate the model and subsequent findings. In fact, such retrospective data are very rare.

#### The parcellation strategy

It is common practice in neuroimaging to use a specific template that defines anatomical regions of the brain. The atlas serves as a common spatial landmark for analysis and interpretation of the data. In our case, it allows us to map and compare brain activity across individuals or groups. Developing a framework for selecting the optimal atlas is essential for defining the relevant ROIs in the study and improving the reproducibility and comparability of research. Indeed, to obtain accurate measurements and meaningful interpretations of the data, it is essential to choose an atlas, whether structural and/or functional, that precisely defines the ROIs suitable for the subsequent analysis. In addition, the development of a framework for selecting the optimal atlas will lay the groundwork for standardizing approaches and guidelines for atlas selection. This will also promote improved reproducibility and facilitate meta-analyses. We propose a machine learning paradigm that provides a sound basis for atlas selection given the underlying clinical question. In our analysis, the most accurate predictions of depth of anesthesia using the proposed BNAs are achieved with the CoCoMac atlas, specifically designed to study cortical regions. This is consistent with GNW and II theories, which suggests that the neural correlates of consciousness are predominantly cortical in nature. In a functional connectivity study, the choice of brain atlas is usually a trade-off between the characterization of brain structure and signal averaging for data reduction and noise reduction. Fine-grained brain areas can capture brain activity descriptions with more functionally-specific regions at the expense of signal-to-noise ratio loss. Fine-grained brain areas also generate high-dimensional input features that are challenging to learn in generic predictive models, a problem known as the curse of dimensionality. Increasing the dataset size will alleviate this problem, and can be achieved through the simulation paradigm proposed in the previous paragraph, or through emerging initiatives such as the PRIMatE Data Exchange [48], which provides free access to an increasing number of fMRI recordings.

### Decoding consciousness

In this study, our focus is on detecting differences directly related to interactions between cortical regions by applying statistical analyses to BNAs categorized by level of consciousness. Unlike alternative methods such as PCA or ICA, BNAs derived from BNs emphasize a significant advantage inherent to the MHA model: a notable improvement in interpretability. Indeed, we identify two main brain networks that account for the ability to decode the state of consciousness. One of them, previously described in the literature, supports the above hypothesized GNW theory. The associated BNAs effectively discriminate between awake and all anesthetic states, which is a strong finding. Additionally, to a lesser extent, these BNAs also distinguish among anesthetic states, underscoring that the use of different anesthetics may alter FC. The other is a new, unnoticed pattern. Specifically, this network is driven by the visual pathway and may be an artifact of our experimental setting, where awake monkeys had open eyes with potential visual stimulation. However, this network is also mainly temporo-parieto-occipital (posterior brain) and could be interpreted with the II theory. Note that in the method of [21] the eye position was tracked during the scans. To clean the discovered networks, we plan to remove windows of saccades or increased visual activity from the awake data. Saccades may selectively generate small motion artifacts (which may have been partially preprocessed) or artificially induce neural coherence in the awake data. Overall, the MHA approach yields few tailored brain networks that can be related to the prominent theoretical frameworks for studying consciousness (the II and GNW theories) and associated BNAs, which promotes interpretability. The model does not rely on biological assumptions about anesthetics and provides results that are insensitive to different anesthetics. Thus, it is reasonable to assume that we are obtaining a general signature of anesthesia-induced loss of consciousness, disentangled from potential markers related to a particular anesthetic effect.

### Potential applications of the proposed framework

The brain is a highly interconnected system consisting of multiple regions that communicate and interact with each other. The proposed framework rethinks the state-of-the-art data-driven strategies. It decomposes the brain signal into brain networks. This provides valuable information about the functional organization of the brain and allows the study of individual differences in brain activity. In fact, each individual has a unique pattern of brain connectivity. Characterizing these individual differences can provide insights into variations in cognitive abilities, behavior, and susceptibility to brain disorders. In addition, understanding individual differences in network connectivity may facilitate the development of personalized treatment approaches, where interventions can be tailored to target specific network dysfunctions in a given individual. The study of network-level properties offers the potential to identify specific biomarkers that can be used for diagnostic purposes, disease monitoring, or prediction of treatment outcomes. Indeed, many neurological and psychiatric disorders are characterized by alterations in brain connectivity. By decomposing the brain signal into networks, we can study how these changes manifest at the network level. For instance, by comparing network properties between healthy individuals and patients, it is possible to identify aberrant connectivity patterns associated with specific disorders. In patients with brain injuries that significantly alter functional connectivity, employing a case-control paradigm can be beneficial. This involves learning a suitable BN decomposition from a healthy cohort that encompasses the desired demographic characteristics while accounting for site effects. Patient data can then be projected onto this BN basis, facilitating the tracking of injury locations and severities. Overall, the proposed approach can lead to a better understanding of the underlying mechanisms of the disorders and potentially help to develop diagnostic or therapeutic strategies. However, clinical validation is needed and opens up a wide avenue for research, including depth of anesthesia monitoring [5, 11], coma characterization [49, 50], and accurate diagnosis of disorders of consciousness in patients [51].

## Supporting information

S1 Fig Brain networks derived from each atlas. The derived brain networks (BNs) consist of sets of unique ROIs represented by their centroids for the CoCoMac (k = 4), DictLearn (k = 6) and CIVMR (k = 7) atlases. When processing data with different atlases, the resulting BNs are not aligned. Therefore, the BNs are sorted using a geometric criterion (*d*_*MoC*_), and the corresponding BN groupings are displayed in columns: A) BNs that are matched in each atlas, and B) BNs from atlases that provide additional brain networks.
(PNG)

S2 Fig Inferred Brain Networks (BNs) and associated BN Activities (BNAs). BNs are inferred from the MHA model using the A) CIVMR (k = 7) and B) DictLearn (k = 6) atlases. Pairwise statistical analysis of the associated BNAs (Statistics) and BNA histograms (Histograms) across different acquisition conditions are presented, in addition to visualizations of the inferred networks (Inferred Networks). The yellow star highlights the BN paired with BN1, as obtained from the CoCoMac atlas. These plots illustrate how clearly awake can be distinguished from all anesthetic states and, to a lesser extent, how all anesthetic states can be distinguished from each other. The legend for the p-value annotation is as follows: **:1.0*e* − 3 < *p* ≤ 1.0*e* − 2, *** : 1.0*e* − 4 < *p* ≤ 1.0*e* − 3, ****:*p* ≤ 1.0*e* − 4.
(PNG)

S3 Fig Inferred Brain Networks (BNs) and associated BN Activities (BNAs) when using a leave-one-subject out strategy. BNs are inferred from the MHA model using the CoCoMac (k = 4) atlas when all data are available (first row) or when one subject is removed during training (second row). For comparison, a pairwise statistical analysis of the associated BNAs and a visualization of these BNAs against the different acquisition conditions are proposed.
(PNG)

S1 Table Summary of the molecular targets of the anesthetics used. Information taken from [15, 17, 52]. Up” means potentiation, and “down” means inhibition. Under the heading “Potassium channels” effects are given for two-pore/inwardly rectifying/voltage-gated channels.
(PDF)

S2 Table Description of the acquisition conditions across monkeys. The data were collected between July 2011 and August 2016 in five rhesus macaques (macaca mulatta), one male (monkey J) and four females (monkeys A, K, L, and R), 5 to 8 kg, 8 to 12 years, either in the awake state or under anesthesia (ketamine, propofol, or sevoflurane). Three monkeys were scanned for each arousal state (awake: monkeys A, K, and J—propofol anesthesia: monkeys K, R, and J—ketamine anesthesia: monkeys K, R, and L—sevoflurane anesthesia: monkeys L, R, and J) with the following repartition.
(PDF)

S3 Table Listing of the networks inferred from the CoCoMac atlas (k = 3). Listing of A) the network 1, B) the network 2, and C) the network 3. The detected GNW areas are depicted in blue, and the associated sensory areas in green.
(PDF)

S4 Table The remaining networks inferred from the CoCoMac (k = 4). Listing of A) the network 2, and B) the network 3. The detected GNW areas are depicted in blue, and the associated sensory areas in green.
(PDF)

S5 Table The networks inferred from the CoCoMac (k = 4) when using a leave-one-subject out strategy. Listing of A) the network matched to BN1, and B) the network matched with BN4. The detected GNW areas are depicted in blue, and the associated sensory areas in green. The +/- signs indicate the addition or deletion of a region in the considered BN.
(PDF)
