# Supplementary figures and images for: Revisiting the standard for modeling functional brain network activity: Application to consciousness
Source: PLoS One. 2024 Dec 16;19(12):e0314598. doi: 10.1371/journal.pone.0314598 (PMC11649112; doi:10.1371/journal.pone.0314598)

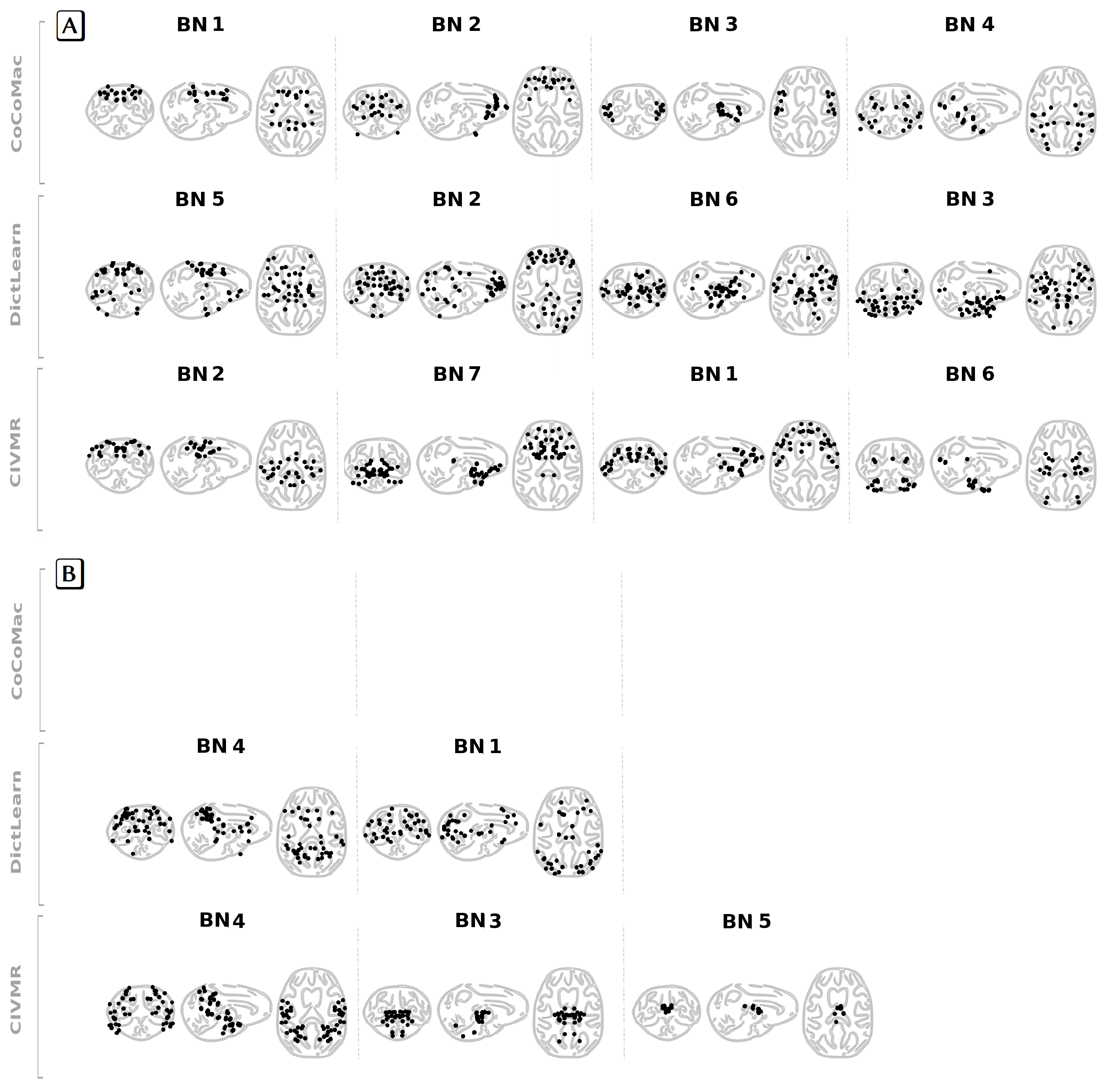

Supplement: S1 Fig — The derived brain networks (BNs) consist of sets of unique ROIs represented by their centroids for the CoCoMac (k = 4), DictLearn (k = 6) and CIVMR (k = 7) atlases. When processing data with different atlases, the resulting BNs are not aligned. Therefore, the BNs are sorted using a geometric criterion (dMoC), and the corresponding BN groupings are displayed in columns: A) BNs that are matched in each atlas, and B) BNs from atlases that provide additional brain networks. (PNG) [file pone.0314598.s001.png]

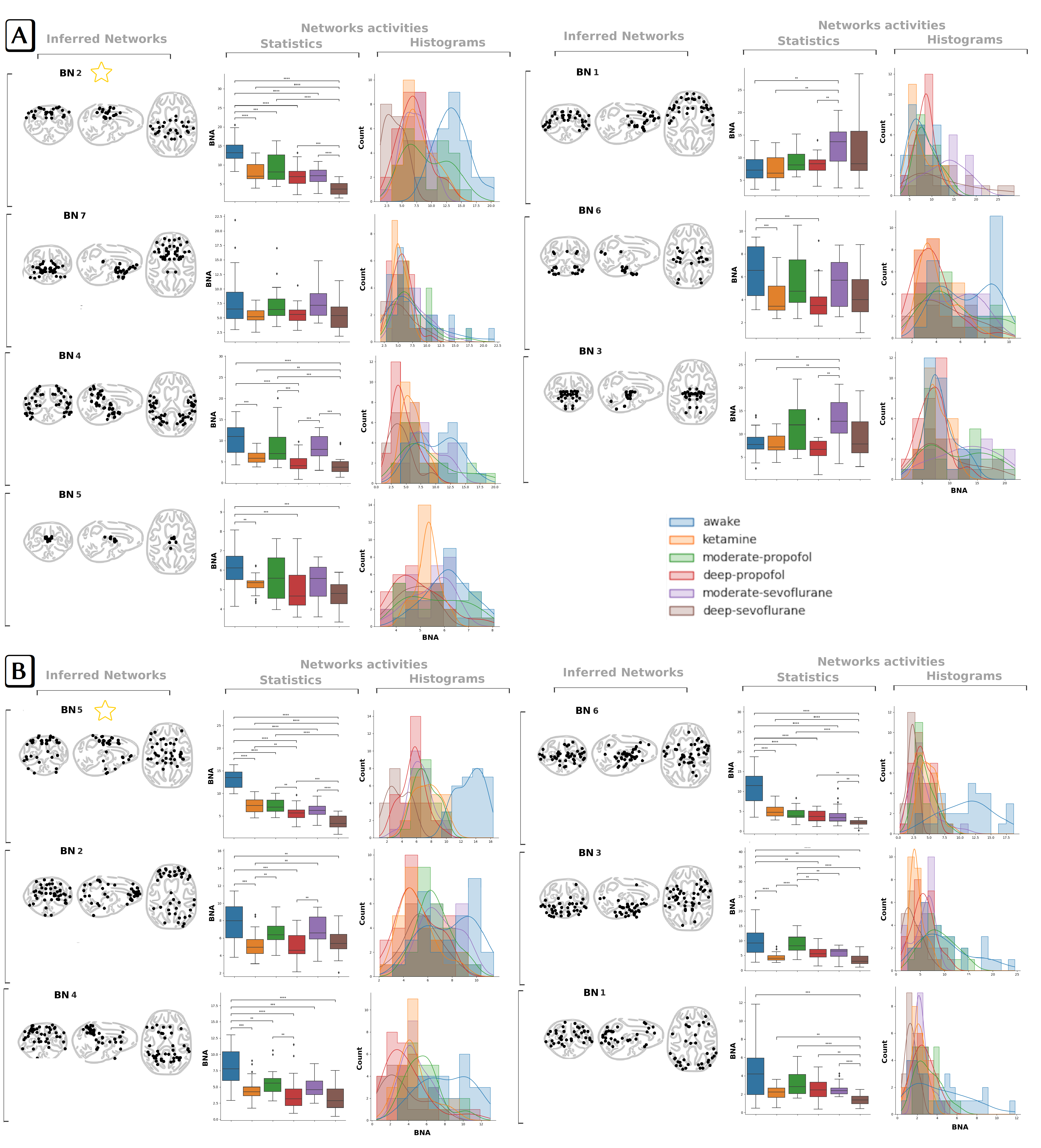

Supplement: S2 Fig — BNs are inferred from the MHA model using the A) CIVMR (k = 7) and B) DictLearn (k = 6) atlases. Pairwise statistical analysis of the associated BNAs (Statistics) and BNA histograms (Histograms) across different acquisition conditions are presented, in addition to visualizations of the inferred networks (Inferred Networks). The yellow star highlights the BN paired with BN1, as obtained from the CoCoMac atlas. These plots illustrate how clearly awake can be distinguished from all anesthetic states and, to a lesser extent, how all anesthetic states can be distinguished from each other. The legend for the p-value annotation is as follows: **:1.0e − 3 < p ≤ 1.0e − 2, *** : 1.0e − 4 < p ≤ 1.0e − 3, ****:p ≤ 1.0e − 4. (PNG) [file pone.0314598.s002.png]

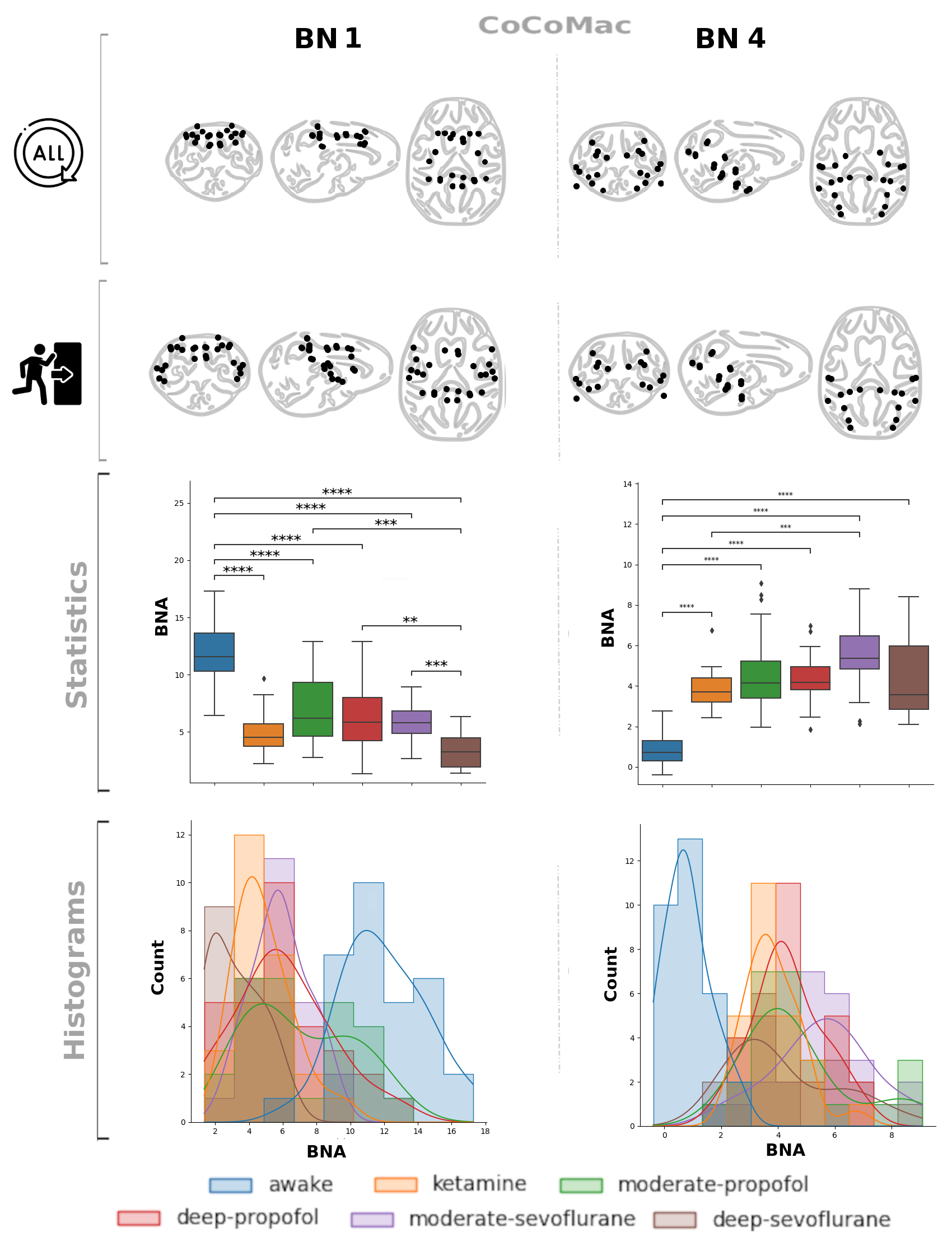

Supplement: S3 Fig — BNs are inferred from the MHA model using the CoCoMac (k = 4) atlas when all data are available (first row) or when one subject is removed during training (second row). For comparison, a pairwise statistical analysis of the associated BNAs and a visualization of these BNAs against the different acquisition conditions are proposed. (PNG) [file pone.0314598.s003.png]
