# Supplementary material for: Revisiting the standard for modeling functional brain network activity: Application to consciousness
Source: PLoS One. 2024 Dec 16;19(12):e0314598. doi: 10.1371/journal.pone.0314598 (PMC11649112; doi:10.1371/journal.pone.0314598)
Supplement: S1 Table — Information taken from [15, 17, 52]. Up” means potentiation, and “down” means inhibition. Under the heading “Potassium channels” effects are given for two-pore/inwardly rectifying/voltage-gated channels. (PDF) [file pone.0314598.s004.pdf]

drug	**GABAa**	**Glycine**	**nAch**	**5-HT**	**AMPA**	**Kainate**	**NMDA**	**Potassium channels**
**Propofol**	up strong	up	down	no effect	down	no effect	down	no effect/no effect/down
**Sevoflurane**	up strong	up strong	down strong	no known effect	down strong	no known effect	down strong	up/no effect/down
**Ketamine**	up	no effect	down strong	up	no effect	no effect	down strong	no known effect/no effect/down
