# Supplementary material for: Revisiting the standard for modeling functional brain network activity: Application to consciousness
Source: PLoS One. 2024 Dec 16;19(12):e0314598. doi: 10.1371/journal.pone.0314598 (PMC11649112; doi:10.1371/journal.pone.0314598)
Supplement: S2 Table — The data were collected between July 2011 and August 2016 in five rhesus macaques (macaca mulatta), one male (monkey J) and four females (monkeys A, K, L, and R), 5 to 8 kg, 8 to 12 years, either in the awake state or under anesthesia (ketamine, propofol, or sevoflurane). Three monkeys were scanned for each arousal state (awake: monkeys A, K, and J—propofol anesthesia: monkeys K, R, and J—ketamine anesthesia: monkeys K, R, and L—sevoflurane anesthesia: monkeys L, R, and J) with the following repartition. (PDF) [file pone.0314598.s005.pdf]

monkey	**awake**	**moderate propofol**	**deep propofol**	**moderate sevoflurane**	**deep sevoflurane**	**ketamine**	
**J**	18	2	8	5	2	-	35
**A**	4	-	-	-	-	-	4
**K**	9	11	10	-	-	8	38
**L**	-	-	-	10	8	7	25
**R**	-	12	12	10	10	10	54
	31	25	30	25	20	25	156
