# Supplementary material for: Revisiting the standard for modeling functional brain network activity: Application to consciousness
Source: PLoS One. 2024 Dec 16;19(12):e0314598. doi: 10.1371/journal.pone.0314598 (PMC11649112; doi:10.1371/journal.pone.0314598)
Supplement: S3 Table — Listing of A) the network 1, B) the network 2, and C) the network 3. The detected GNW areas are depicted in blue, and the associated sensory areas in green. (PDF) [file pone.0314598.s006.pdf]

	name	hemi	location
**CCp**	posterior cingulate cortex	left, right	cingulate cortex
**CCa**	anterior cingulate cortex	left, right	cingulate cortex
**S1**	primary somatosensory cortex	left, right	parietal cortex
**PCi**	inferior parietal cortex	left, right	parietal cortex
**PCm**	medial parietal cortex	left, right	parietal cortex
**PCip**	intraparietal cortex	left, right	parietal cortex
**PCs**	superior parietal cortex	left, right	parietal cortex
**M1**	primary motor cortex	left, right	frontal cortex
**FEF**	frontal eye field	left, right	frontal cortex
**PMCm**	medial premotor cortex	left, right	frontal cortex
**PMCdl**	dorsolateral premotor cortex	left, right	frontal cortex
**PMCvl**	ventrolateral premotor cortex	left, right	frontal cortex
**PFCdm**	dorsomedial prefrontal cortex	left, right	frontal cortex
**G**	gustatory cortex	left, right	gustatory cortex

(A)

	name	hemi	location
**TCpol**	temporal polar	left, right	temporal cortex
**Amyg**	amygdala	right	temporal cortex
**PFCoi**	orbitoinferior prefrontal cortex	left, right	frontal cortex
**PFCom**	orbitomedial prefrontal cortex	left, right	frontal cortex
**PFCol**	orbitolateral prefrontal cortex	left, right	frontal cortex
**PFCpol**	prefrontal polar cortex	left, right	frontal cortex
**PFCvl**	ventrolateral prefrontal cortex	left, right	frontal cortex
**PFCm**	medial prefrontal cortex	left, right	frontal cortex
**PFCcl**	centrolateral prefrontal cortex	left, right	frontal cortex
**PFCdl**	dorsolateral prefrontal cortex	left, right	frontal cortex
**CCs**	subgenual cingulate cortex	left, right	cingulate cortex

(B)

	name	hemi	location
**TCs**	superior temporal cortex	left, right	temporal cortex
**Ia**	anterior insula	left, right	insular cortex
**Ip**	posterior insula	left, right	insular cortex
**A2**	secondary auditory cortex	left, right	temporal cortex
**S2**	secondary somatosensory cortex	left, right	parietal cortex
**A1**	primary auditory cortex	left, right	temporal cortex
**TCc**	central temporal cortex	left, right	temporal cortex
**CCr**	retrosplenial cingulate cortex	right	cingulate cortex
**TCi**	inferior temporal	left	temporal cortex

(C)
