# Supplementary material for: Revisiting the standard for modeling functional brain network activity: Application to consciousness
Source: PLoS One. 2024 Dec 16;19(12):e0314598. doi: 10.1371/journal.pone.0314598 (PMC11649112; doi:10.1371/journal.pone.0314598)
Supplement: S4 Table — Listing of A) the network 2, and B) the network 3. The detected GNW areas are depicted in blue, and the associated sensory areas in green. (PDF) [file pone.0314598.s007.pdf]

| | name | hemi | location |
|---|---|---|---|
| **TCpol** | temporal polar | left, right | temporal cortex |
| **PFCoi** | orbitoinferior prefrontal cortex | left, right | frontal cortex |
| **PFCom** | orbitomedial prefrontal cortex | left, right | frontal cortex |
| **PFCol** | orbitolateral prefrontal cortex | left, right | frontal cortex |
| **PFCpol** | prefrontal polar cortex | left, right | frontal cortex |
| **PFCvl** | ventrolateral prefrontal cortex | left, right | frontal cortex |
| **PFCm** | medial prefrontal cortex | left, right | frontal cortex |
| **PFCcl** | centrolateral prefrontal cortex | left, right | frontal cortex |
| **PFCdm** | dorsomedial prefrontal cortex | left, right | frontal cortex |
| **PFCdl** | dorsolateral prefrontal cortex | left, right | frontal cortex |
| **CCs** | subgenual cingulate cortex | left, right | cingulate cortex |

(A)

| | name | hemi | location |
|---|---|---|---|
| **TCs** | superior temporal cortex | left, right | temporal cortex |
| **A1** | primary auditory cortex | left, right | temporal cortex |
| **A2** | secondary auditory cortex | left, right | temporal cortex |
| **G** | gustatory cortex | left, right | gustatory cortex |
| **PMCvl** | ventrolateral premotor cortex | left, right | frontal cortex |
| **Ip** | posterior insula | left, right | insular cortex |
| **Ia** | anterior insula | left, right | insular cortex |
| **S2** | secondary somatosensory cortex | left, right | parietal cortex |

(B)
