# Supplementary material for: Revisiting the standard for modeling functional brain network activity: Application to consciousness
Source: PLoS One. 2024 Dec 16;19(12):e0314598. doi: 10.1371/journal.pone.0314598 (PMC11649112; doi:10.1371/journal.pone.0314598)
Supplement: S5 Table — Listing of A) the network matched to BN1, and B) the network matched with BN4. The detected GNW areas are depicted in blue, and the associated sensory areas in green. The +/- signs indicate the addition or deletion of a region in the considered BN. (PDF) [file pone.0314598.s008.pdf]

	name	hemi	location
**CCp**	posterior cingulate cortex	left, right	cingulate cortex
**CCa**	anterior cingulate cortex	left, right	cingulate cortex
**S1**	primary somatosensory cortex	left, right	parietal cortex
**PCi**	inferior parietal cortex	left, right	parietal cortex
**PCm**	medial parietal cortex	left, right	parietal cortex
**PCip**	intraparietal cortex	left, right	parietal cortex
**PCs**	superior parietal cortex	left, right	parietal cortex
**M1**	primary motor cortex	left, right	frontal cortex
**- FEF**	frontal eye field	left, right	frontal cortex
**PMCm**	medial premotor cortex	left, right	frontal cortex
**- PMCdl**	dorsolateral premotor cortex	left, right	frontal cortex
**+ PMCvl**	ventrolateral premotor cortex	left, right	frontal cortex
**+ Ip**	posterior insula	left, right	insular cortex
**+ A1**	primary auditory cortex	left, right	temporal cortex
**+ A2**	secondary auditory cortex	left, right	temporal cortex
**+ S2**	secondary somatosensory cortex	left, right	parietal cortex

(A)

	name	hemi	location
**- Amyg**	amygdala	left, right	temporal cortex
**TCc**	central temporal cortex	left, right	temporal cortex
**TCi**	inferior temporal	left, right	temporal cortex
**PHC**	parahippocampal cortex	left, right	temporal cortex
**- HC**	hippocampus	left, right	temporal cortex
**TCv**	ventral temporal cortex	left, right	temporal cortex
**VACv**	anterior visual area (ventral)	left, right	occipital cortex
**V2**	visual area 2	left, right	occipital cortex
**VACd**	anterior visual area (dorsal)	left, right	occipital cortex
**V1**	visual area 1	left, right	occipital cortex
**CCr**	retrosplenial cingulate cortex	left, right	cingulate cortex

(B)
